# Specialist physician perspectives on clinical decision support to address secondary vaccine hesitancy

**DOI:** 10.1016/j.jacig.2025.100636

**Published:** 2025-12-22

**Authors:** Anjali Nemorin, Dylan T. Norton, Chloe V. Green, Michelle S. Jerry, Alysse G. Wurcel, Kimberly G. Blumenthal

**Affiliations:** a Division of Infectious Diseases, Massachusetts General Hospital, Boston, Mass; b Infection Control, Massachusetts General Hospital, Boston, Mass; c Division of Rheumatology, Allergy, and Immunology, Department of Medicine, Massachusetts General Hospital, Boston, Mass; d Rheumatology and Allergy Clinical Epidemiology Research Center, The Mongan Institute, Boston, Mass; e Section of General Internal Medicine, Boston Medical Center, Boston, Mass; f Harvard Medical School, Boston, Mass

**Keywords:** Allergy, adverse event following immunization, adverse drug event, adverse reaction, infectious disease specialist, allergist, vaccine reactions, interview, CDS system, CDS tool

## Abstract

**Background:**

Vaccines are an evidence-based intervention that mitigates the impact of infections, yet many Americans indicate hesitancy toward receiving vaccines. One of the most common reasons for this is the potential for experiencing adverse reactions when receiving vaccines. The current literature shows that clinical decision support (CDS) tools have been utilized to improve vaccine coverage.

**Objective:**

Our aim was to assess specialist physician experiences, practices, and levels of comfort with evaluating adverse and allergic reactions to vaccines, as well as with addressing secondary vaccine hesitancy, using CDS tools.

**Methods:**

Researchers conducted 10 semistructured interviews with physicians in Mass General Brigham’s Infectious Diseases and Allergy/Immunology units. The interview guide consisted of 11 questions divided into 4 sections: vaccine conversations, vaccine allergies/reactions, CDS, and structural facilitators/barriers. The interview responses were evaluated by using rapid thematic analysis.

**Results:**

Specialist physicians were generally comfortable talking to patients about vaccine hesitancy but were also open to CDS tools that effectively and efficiently contribute to improved conversations around secondary vaccine hesitancy to increase vaccine uptake. Desired CDS tool features include sharing educational visuals and videos with patients. The risks involve operational delays and lack of real-time data and accountability structures. Clinic modifications such as longer appointment times or group appointments were considered as other ways to help address vaccine hesitancy.

**Conclusion:**

Specialist physicians expressed interest in using CDS tools to improve vaccine-related conversations with their patients. Future CDS tools must account for timely vaccine information and workflow efficiency issues. Future research should include generalist physicians, other health care team members, and patients.

## Introduction

Vaccines are universally heralded as a key public health initiative necessary to prevent deaths from infections. The coronavirus disease 2019 (COVID-19) pandemic demonstrated vaccines’ importance but fueled misinformation about vaccine safety. Primary vaccine hesitancy is when a person is reluctant to be vaccinated with a vaccine that he or she has never received.^1^ Less commonly discussed is *secondary vaccine hesitancy*, a broad term that can refer to a person being reluctant to get a vaccine again, even if he or she has received vaccines in the past. This can even stretch to reluctance to receive other vaccines never previously taken, often because of fear of experiencing adverse reactions such as those encountered previously. Although primary vaccine hesitancy has been studied extensively,^1^, ^2^, ^3^ the data on the epidemiology of and/or clinical approaches to secondary vaccine hesitancy are limited.

Although people commonly report adverse reactions to vaccines, true vaccine allergies that represent contraindications to repeated vaccination (eg, anaphylaxis, angioedema) are rare.^4^ For all vaccines, anaphylaxis was reported at a rate of 1.31 per million doses,^5^ and cutaneous symptoms (eg, urticaria, pruritus, angioedema) were reported in 1.9% of first doses of the COVID-19 mRNA vaccine.^6^

Patients’ reports of vaccine allergies often represent a misclassification. For example, people report that the influenza vaccine “gave them the flu” or that vaccines caused arm pain. However, these are often expected adverse reactions or side effects.^7^ This misclassification indicates a lack of education and/or communication between patients and health care teams regarding risks, benefits, and expectations of vaccination, and it leads to reduced uptake.

The current literature shows that clinical decision support (CDS)—software designed to be a direct aid to clinical-decision making with patient-specific assessments^8^—has been utilized to improve vaccine coverage, from automatically sending patients and providers reminders when vaccines are due to automatically queueing vaccine orders for upcoming patient visits.^9^^,^^10^

The objective of this study was to evaluate specialist physician perspectives on barriers to and facilitators of addressing secondary vaccine hesitancy. Within this goal, we assessed physicians’ views on using CDS to support accurate assessment and documentation of vaccine allergy, reduce vaccine hesitancy, increase vaccine confidence, and increase vaccine uptake.

Following a formative review of the literature on topics of secondary vaccine hesitancy and CDS tools, we used the Consolidated Framework for Implementation Research (CFIR) 2.0 domains as a scaffold for development of our interview guide,^11^ defining the evidence-based intervention within the CFIR framework as vaccines, and the implementation strategy as support for increased access to vaccines as CDS tools (see the Supplementary Material in the Online Repository at www.jaci-global.org). Interviews lasting 20 to 45 minutes were conducted over Zoom by trained research staff from February through April 2025. Ethical approval was obtained from the Mass General Brigham institutional review board, and informed consent was obtained verbally from all participants, who were recruited via purposive, convenience sampling. We used Rapid Thematic Analysis (RTA), a streamlined qualitative research method that is CFIR based and deductive. We used 2 coders, one coding in real time and the other coding using the audio recording.^12^ To ensure timeliness and accuracy, both interviewers performed RTA within 72 hours. The initial codes were developed deductively and reviewed using a team approach to ensure consistency. The team met several times to resolve discrepancies and achieve consensus on key themes according to CFIR 2.0 constructs.

## Results and discussion

Of the 25 physicians recruited, 10 (ie, 3 infectious disease doctors and 7 allergists/immunologists) completed interviews (Table I). RTA revealed 3 major themes, as follows.

**Table I:** Specialist physician perspectives on using CDS to address secondary vaccine hesitancy

Theme	Physician perspectives
Desired features and potential benefits of CDS to address secondary vaccine hesitancy	“We have several dot phrases that were helpful. One was a dot phrase evaluation note…. It would dot phrase in a list of medical information and details that we should be asking in order to make sure we were thoroughly evaluating that patient… depending on how those questions were answered by the patient offered some recommendations for what it might mean for mild, moderate, or severe reactions.” (physician 3 [an allergist]) “I think [CDS] would need [*sic*] to include like if there’s a kind of a flowchart or a flowsheet in the same way that we have for the penicillin allergy pathway at [Massachusetts General Hospital], that kind of tells you, OK this was the reaction, and these are the things you can do next.” (physician 2 [an infectious disease physician]) “A nice video research summary that I could put in the patient’s chart… the [American Academy of Allergy, Asthma & Immunology] has nice pages about food sensitivity testing, a little description, and a video of an allergist saying their shpiel very quick… I can’t have these 5- to 10-minute conversations times 3 issues in 1 visit.” (physician 8 [an allergist])
Potential barriers and risks of using CDS tools to address secondary vaccine hesitancy	“I hesitate to add stuff to the primary care doctor’s visit and time, so it would have to be implemented in a way that is time-neutral or a time saver… to access a thing to look at and then guide me through a discussion might take me 5 to 10 minutes, I’m not sure I would utilize that.” (physician 5 [an allergist]) “I suppose if you had clicked on the care gap where it said that, you know, they were due for the influenza vaccine and there was some information or resources there that might help. But otherwise I don't know the best way. I would not recommend, like, pop-up things. Everyone hates those and they kind of interrupt.” (physician 2 [an infectious disease physician]) “[Advisory Committee on Immunization Practices information] is accurate and complete and useful… but over the past decade they’ve changed the recommendations… a bunch of times they come out with new vaccines, so sometimes it was confusing, and I’d have to review it… it is evolving, not consistent.” (physician 5 [an allergist]) “We’re working with some of these national sites across the country to import our code for Epic… Even if we use Epic and [the University of California San Francisco] uses Epic, they have to develop their own. There’s no way for Epic to share among sites unless they’re in the same network, so it’s ridiculous.” (physician 1 [an infectious disease physician])
Clinic modifications to address secondary vaccine hesitancy	“I learned [that] at [Massachusetts General Hospital] they do group visits for certain preventable diseases in primary care. It actually might be really interesting to do a group visit with vaccine-hesitant people to allow you to present information for like-minded people.” (physician 7 [an allergist]) “If people are hesitant to get something, they aren’t going to come for an extra visit… if the rates are really going up and it becomes a longer discussion to convince people to get a vaccine, then I think the clinic slots should be extended.” (physician 6 [an infectious disease physician])

### Theme 1: Desired features and potential benefits of CDS tools to address vaccine hesitancy

Many physicians indicated that they actively utilize their personally created dot phrases (precreated phrases summoned into a note using a period) to navigate vaccine decision making, suggesting that better dot phrases could improve discussions with patients about vaccine hesitancy. In reference to COVID-19, one allergist (physician 3) said that a helpful CDS tool was a “dot phrase evaluation note” prompting recommendations based on the severity of the patient’s reaction. Other physicians emphasized the need for CDS tools to clearly and efficiently communicate information visually. Another physician (physician 2 [an infectious disease physician]) suggested that new tools could model the flowcharts and flowsheets used for their existing penicillin allergy pathways. Time was an issue raised by 1 physician as it relates to potential use of a video tool that could support counseling: “a nice video research summary that I could put in the patient’s chart… a little description, and a video of an allergist saying their spiel very quick” (physician 8 [an allergist]).

### Theme 2: Potential barriers and risks of a CDS tool to address vaccine hesitancy

On the flip side, some specialists thought that CDS tools would slow clinic operations down. One allergist (physician 5) said, “it would have to be implemented in a way that is time-neutral or a time saver… to access a thing to look at and then guide me through a discussion might take me 5 to 10 minutes, I’m not sure I would utilize that.” Some physicians wondered how the CDS tools would continue to be updated given the constant changes in vaccine guidance: “[Advisory Committee on Immunization Practices information] is accurate and complete and useful… but over the past decade they’ve changed the recommendations… so sometimes it was confusing, and I’d have to review it,” (physician 5 [an allergist]). In the case of national infectious disease management, physicians worry about CDS not being able to communicate information across institutions: “We’re working with some of these national sites across the country to import our code for Epic [a common US electronic health record]….There’s no way for Epic to share among sites unless they’re in the same network” (physician 1 [an infectious disease physician]).

### Theme 3: Modifying clinic operations to address secondary vaccine hesitancy

To counter time constraint concerns, some physicians advocated for a workflow adjustment to improve the efficiency of answering patients’ questions about vaccine risks. Some suggested new appointment mechanisms such as group visits or appointments dedicated entirely to vaccine discussions. One allergist (physician 7) said, “It actually might be really interesting to do a group visit with vaccine-hesitant people to allow you to present information for like-minded people.” Others emphasized the need to build extra time into their visits to allow the opportunity to counsel their patients on vaccine hesitancy: “If people are hesitant to get something, they aren’t going to come for an extra visit… if the rates are really going up and it becomes a longer discussion to convince people to get a vaccine, then I think the clinic slots should be extended,” (physician 6 [an infectious disease physician]).

This qualitative study of infectious disease physicians and allergists can support future interventions aimed at facilitating conversations about secondary vaccine hesitancy. Vaccine hesitancy has been named one of the top 10 public health threats by the World Health Organization.^4^ Reductions in vaccine uptake pose pervasive ripple effects such as vaccine refusal, vaccine delay, incomplete vaccination, and loss of herd immunity.

CDS creation will need to consider practical suggestions for improving conversations about secondary vaccine hesitancy, such as incorporating multimedia education tools into patient records and for patient distribution. This avenue needs to consider the range of modes and materials, the availability of computers to share these tools, and accountability regarding who will update them. CDS, when designed poorly, can be ignored or overridden, present clinically inappropriate information, and cause alert fatigue for clinicians.^13^^,^^14^ Also, when discussing CDS tools in the interview guide, the research team found that many physicians did not realize that some of the actions they took clinically, such as use of EHR dot phrases, are considered CDS. To further understand clinician opinions on CDS tools, future research should establish a consistent, accurate definition for CDS.

Limited time for clinical interactions is a barrier to effective vaccine counseling. Clinicians may refer patients with vaccine concerns to allergists and other specialists for further evaluation.^15^ Allergies entered in patient charts are “rarely edited or removed,” resulting in growing allergy lists that wrongfully delay and prevent vaccinations because providers cannot confidently recommend vaccines and lack the tools to further investigate efficiently.^16^ The time and resources of allergists and other specialists’ are then occupied by revising allergies in the patient’s charts. How many people with vaccine allergies are referred to allergists is not known, but this should be a service offered and advertised. Additionally, when allergists are seeing patients for other allergies (medicine, foods, environmental allergens), that encounter should be used to discuss vaccine allergies, where applicable. The theme “clinic modifications to address secondary vaccine hesitancy” included clinic redesign options such as group visits, which could augment the effectiveness of CDS. Such visits could build a community of people empowering each other, or it could potentially fuel anxiety and hesitancy.

This study was limited by a small scope of physician specialties, covering only allergists and infectious disease physicians practicing at a single health system in the northeastern United States. Although we initially aimed to include primary care physicians, pediatricians, and other health care professionals who address vaccine concerns in routine outpatient visits, study funding was unexpectedly halted. We acknowledge that CDS is only one of many possible solutions to addressing secondary vaccine hesitancy and that CDS is most effective when part of a multicomponent implementation package utilizing different strategies and interventions.^17^ Despite these limitations, this work is a first step in the process of building research with key stakeholders aimed to support discussions about secondary vaccine hesitancy and CDS tool use. Future research should prioritize inclusion of primary care providers, whose role positions them to influence vaccine decision making on a broader scale, as well as the perspectives and needs of vaccine-hesitant patients.

**Key messages**

• Secondary vaccine hesitancy is common but underresearched; reactions are often misclassified as allergies.
• Physicians see value in CDS; time, consistency, and accuracy are concerns.
• Enhanced dot phrases and group visits may improve vaccine counseling.

## Disclosure statement

Supported in part by a cooperative agreement from the US Centers for Disease Control and Prevention (no. CK22-2203 [principal investigator: Erica S. Shenoy, PhD, MD]), the 10.13039/100000002National Institutes of Health (to K.G.B.), and the Agency for Healthcare Research and Quality (to K.G.B.). The funders had no role in the design and conduct of the study; collection, management, analysis, and interpretation of the data; preparation, review, or approval of the article; and decision to submit the article for publication.

Disclosure of potential conflict of interest: K. G. Blumenthal reports royalties from UpToDate, during the conduct of the study. The rest of the authors declare that they have no relevant conflicts of interest.

Data sharing statement: Individual-level data will not be shared. Investigators interested in using de-identified or aggregate data for their own projects are invited to submit a formal request to the research team.

Access to data and data analysis: All authors except Alysse G. Wurcel had full access to all the data in the study and take responsibility for the integrity of the data and accuracy of the data analysis.

Declaration of generative artificial intelligence and artificial intelligence–assisted technologies in the writing process. During the preparation of this work the author(s) used ChatGPT to increase conciseness and clarity. After using this tool/service, the author(s) reviewed and edited the content as needed and take full responsibility for the content of the publication.
