# Supplementary material for: Specialist physician perspectives on clinical decision support to address secondary vaccine hesitancy
Source: J Allergy Clin Immunol Glob. 2025 Dec 22;5(2):100636. doi: 10.1016/j.jacig.2025.100636 (PMC12808990; doi:10.1016/j.jacig.2025.100636)
Supplement: Supplementary Material [file mmc1.docx]

# In-Depth Interview Guide – Clinicians

Interviewer must fill in the following details:

Participant ID ____________ Interview Date (MM/DD/YYYY) _____________

**Introduction Script**

My name is (INTERVIEWER NAME). I am a [role] at Massachusetts General Hospital. [If applicable, introduce accompanying research assistants]. Thank you on behalf of my team for agreeing to be part of this interview. We are asking you to participate in this interview because you may treat patients with a reported allergy or adverse reaction to one or many vaccines. I would like to ask you questions about your thoughts about caring for these patients. There are no “right” or “wrong” answers to these questions. To make sure that I have an accurate report of information from your interview, I will use a digital audio recorder. Do I have your permission to audio record this interview?

YES NO

*Audio recorder turned ON (circle if yes)*

If you do not feel comfortable answering a question, it is okay for you to tell me that you do not wish to answer. If there’s a question that you would prefer not to answer, you may simply say “pass”. You can also choose to stop this interview at any time and for any reason.

Before we begin, do you have any questions?

*Refer to the study fact sheet to answer questions.*

Great. Can I ask you to respond ‘yes’ to confirm that you would like to take part in this interview?

Verbal Consent Conducted by: _____________________________________

(Interviewer’s Signature)

Time Interview Started: _______ AM / PM

Time Interview Ended: _______ AM / PM

### Vaccine Conversations

1. To begin with, can you walk me through the process of deciding if a patient you are seeing should be offered a vaccine?
   1. Who else on the clinical team discusses vaccine with patients (for example, nurses, MAs, or pharmacists?
   2. What resources do you use to assist you in deciding if a patient needs a vaccine? (Patients? EHR? Other staff? National guidelines?)
   3. How accurate, complete, or useful to you do you think that information is?

###### Notes:

_______________________________________________________

________________________________________________________

________________________________________________________________________________________________________________________________________________________________________________________________________________________________

________________________________________________________________________________________________________________________________________________________________________

1. How do you typically discuss expected reactions or side effects from vaccines with your patients?

###### Notes:

_______________________________________________________

________________________________________________________

________________________________________________________________________________________________________________________________________________________________________________________________________________________________

1. What do you hear from your colleagues about vaccines and vaccine reactions and/or allergies?
   1. [If they *don’t* discuss conversations about vaccines or vaccine reactions]: Why do you think that is?
   2. Have you ever been part of a conversation with your colleagues or overheard any of your colleagues discuss concerns about medical liability if a patient reports a side effect from a vaccine?

###### Notes:

_______________________________________________________

________________________________________________________

_______________________________________________________________________________________________________________________________________________________________________________________________________________________________________________________________________________________

### Vaccine Allergies/Reactions

1. What types of reactions would prompt you to add a vaccine allergy to a patient’s list?
   1. Can you share experience about a time when you had a patient tell you they had an allergy to a vaccine?
   2. How do you distinguish between expected reactions versus allergies?
   3. How comfortable do you feel evaluating someone’s report of a vaccine allergy? (For example, if someone reports a history of fainting after getting a vaccine, how would you evaluate that when they come in for a visit?)

###### Notes:

_______________________________________________________

________________________________________________________

________________________________________________________________________________________________________________________________________________________________________________________________________________________________

Now we’re going to talk a bit more about vaccine allergies or vaccine reactions and I want to clarify the kind of information I want you to focus on in this set of questions.

You might have experienced some patients who don’t want to get vaccinated at all, which is an example of primary vaccine hesitancy.

There are *also* patients who have previously gotten vaccines and have had reactions to them which makes them not want to get future vaccines. That is an example of *secondary* vaccine hesitancy. People can express secondary vaccine hesitancy with the same vaccine that they previously had a reaction to, or towards a vaccine that they haven’t received.

1. What is your comfort level in discussing vaccines with people who have secondary vaccine hesitancy?
2. How comfortable are you in recommending one type of vaccine to people who report a specific allergy or reaction to a **different** vaccine? For example, recommending the flu vaccine to a patient who reports a reaction from the COVID-19 vaccine.
3. How comfortable are you in recommending one type of vaccine to people **who report a specific allergy or reaction to that vaccine**? For example, recommending the flu vaccine to a patient who reports that the flu vaccine ‘gave them the flu’?
4. What would increase your comfort/knowledge?
5. What would make you feel comfortable deleting allergies or overriding allergy alerts in a patient’s electronic medical record?

###### Notes:

_______________________________________________________

________________________________________________________

________________________________________________________________________________________________________________________________________________________________________________________________________________________________________________________________________________________

### CDS

Now I want to talk about tools in electronic health records that help clinicians make decisions, otherwise known as “clinical decision support tools”.

1. What is your experience of using EHR tools such as dot phrases, flowsheets, reminders, best practice advisory, order sets?
   1. How do clinical decision support tools help you talk with patients who have secondary vaccine hesitancy about getting future vaccines?

###### Notes:

_______________________________________________________

________________________________________________________

________________________________________________________________________________________________________________________________________________________________________________________________________________________________

1. If you were to have a tool in Epic that could assist you with documenting vaccine hesitancy, vaccine allergy, or vaccine counseling, what would it need to include?
   1. Can you think of any tools specific to secondary vaccine hesitancy that you would want to have?

###### Notes:

_______________________________________________________

________________________________________________________

________________________________________________________________________________________________________________________________________________________________________________________________________________________________________________________________________________________________________________________________________________

1. How worried are you about the medical liability of using a clinical decision support tool to assist you with vaccinating patients?
2. For example, if the tool said that the person who reported a headache to COVID vaccine could safely get a COVID vaccine, would you feel legally protected in recommending the vaccine?
3. Are there other examples of adverse reactions you would feel more or less comfortable being supported by a clinical decision support tool for?
4. What would make you feel less concerned about liability?
5. How would your comfort level change if you knew that our CDSS was reviewed by the legal team at MGB?

###### Notes:

_______________________________________________________

________________________________________________________

________________________________________________________________________________________________________________________________________________________________________________________________________________________________

1. What alternatives to clinical decision support tools do you think would work better to improve your experience with addressing vaccine reactions?

###### Notes:

_______________________________________________________

________________________________________________________

________________________________________________________________________________________________________________________________________________________________________________________________________________________________________________________________________________________________________________________________________________

### Other/Structural

1. What kinds of workflow changes are necessary to accommodate implementing a clinical decision support tool for secondary vaccine hesitancy?
   1. How worried are you about increased vaccines tying up the clinical staff and preventing flow through clinic?
   2. What about changes to scheduling options such as increased clinic hours of operation, or more time per visit, or visits specifically for vaccine hesitancy counseling?

###### Notes:

_______________________________________________________

________________________________________________________

________________________________________________________________________________________________________________________________________________________________________________________________________________________________

1. What kind of patient support would be necessary to increase patient engagement in getting vaccines if they report a history of vaccine reactions?
   1. Some barriers may include time, transportation, finances, scheduling, etc.

###### Notes:

_______________________________________________________

________________________________________________________

________________________________________________________________________________________________________________________________________________________________________

Thank you for your time. I just have a short survey to capture some basic demographic details about you, so I will now turn off the recorder now.

# Sociodemographic Questionnaire

Check the box/write in the answer that fits you best in each question. Thank you for your participation. This information will be used to better understand the characteristics and representativeness of our sample.

Participant Name ____________

Interview Date (MM/DD/YYYY) _____________

**1. Age**

What is your age?

- 18–24
- 25–34
- 35–44
- 45–54
- 55–64
- 65 and older
- Prefer not to answer
- **Other** (Please specify): ______________

**2. Gender**

Which gender best describes you?

- Man
- Non-binary
- Woman
- Prefer not to answer
- Prefer to self-describe: ______________

**3. Race**

What is your race? (Select all that apply)

- American Indian or Alaska Native
- Asian
- Black or African American
- Middle Eastern or North African
- Native American or Indigenous
- Native Hawaiian or Other Pacific Islander
- White
- Prefer not to answer
- Prefer to self-describe: ______________

**4. Ethnicity**

What is your ethnicity?

- Hispanic or Latino
- Non-Hispanic or Non-Latino
- Prefer not to answer
- Prefer to self-describe: ______________

**5. Years in Practice**

How many years have you been a practicing clinician?

- Less than 1 year
- 1-3 years
- 4-6 years
- 7-9 years
- 10-15 years
- 15-20 years
- 20+ years
- Prefer not to answer
- **Other** (Please specify): ______________
